# Chronic Endometritis and Recurrent Pregnancy Loss: A review of evidence and underlying mechanisms

**DOI:** 10.5935/1518-0557.20250176

**Published:** 2026

**Authors:** Fernanda Chaves Capanema Álvares, Frederico Timm Rodrigues de Sousa, Elaine Cristina Fontes de Oliveira

**Affiliations:** 1 Division of Human Reproduction, Hospital das Clínicas, Federal University of Minas Gerais, Belo Horizonte, Minas Gerais, Brazil

**Keywords:** clinical protocols, chronic endometritis, hysteroscopy, recurrent pregnancy loss, treatment

## Abstract

This narrative review aims to evaluate the relationship between chronic endometritis (CE) and recurrent pregnancy loss (RPL). A comprehensive, non-systematic search on the association between RPL and CE was conducted in the PubMed, Cochrane, and SciELO databases, considering its pathophysiological mechanisms, diagnosis and pregnancy outcomes after treatment. The following terms were used as keywords: (“chronic endometritis” OR “endometrial inflammation” OR “subclinical endometritis”) AND (“recurrent pregnancy loss” OR “recurrent miscarriage” OR “pregnancy outcomes”). Diagnostic hysteroscopy can be useful for identifying CE based on visual findings (e.g., edema and endometrial micropolyps). However, specificity and sensitivity vary, and it is ideally complemented by biopsy. The identification of plasma cells using the CD-138 marker is the most accurate method for diagnosing CE. Standardization is still needed for the number of plasma cells considered diagnostic and for the quality of CD-138. Tests such as next-generation sequencing and real-time PCR can identify microorganisms and aid in the development of appropriate treatments. Antibiotic regimens have shown efficacy in reducing CE and have a positive impact on pregnancy outcomes in women with RPL. In conclusion, CE appears to be a significant but often underdiagnosed contributor to RPL. Advancements in diagnostic techniques have improved the accuracy of CE identification. Evidence suggests that effective antibiotic treatment not only resolves CE but also enhances pregnancy outcomes in affected women.

## INTRODUCTION

Recurrent pregnancy loss (RPL) is classically defined as the loss of three consecutive pregnancies before 20 weeks of gestation (Rai & Regan, 2006). The guidelines for definition, diagnostic testing, and treatment vary among different societies, with investigation of causal factors currently recommended from the second abortion onwards. (Practice Committee of the ASRM, 2012; Ferriani *et al*., 2018; ESHRE Guideline Group on RPL *et al*., 2023; Regan *et al*., 2023). The American Society for Reproductive Medicine (ASRM) considers only clinical pregnancies (those diagnosed by ultrasound or histopathological examination) for diagnosis, excluding molar and ectopic pregnancies (Practice Committee of the ASRM, 2012). Meanwhile, the Royal College of Obstetricians and Gynaecologists (RCOG) and the European Society of Human Reproduction and Embryology (ESHRE) include biochemical pregnancies as a pregnancy criterion (ESHRE Guideline Group on RPL *et al*., 2023; Regan *et al*., 2023). The most recent definition encompasses both spontaneous pregnancies and those following assisted reproductive technologies (ART), excluding molar and ectopic pregnancies as well as implantation failures (Tulandi & Al-Fozan, 2024).

The prevalence of RPL varies depending on the early recognition of pregnancy and the criteria used for its definition (Practice Committee of the ASRM, 2012; Ferriani *et al*., 2018; ESHRE Guideline Group on RPL *et al*., 2023; Regan *et al*., 2023; Dimitriadis *et al*., 2020). It affects approximately 3% of couples attempting to conceive, considering at least two losses, and about 1% when there are three or more losses (Rai & Regan, 2006). The prevalence of abnormal test results for RPL does not differ between couples with two or three losses, and it is currently recommended to investigate causal factors after two miscarriages (van Dijk *et al*., 2020).

Known causes of (RPL) include genetic or chromosomal abnormalities in the parents or embryo, maternal immunological disorders, endocrine disorders, acquired thrombophilias, environmental factors, infectious causes (endometritis), and uterine anatomical abnormalities (Christiansen *et al*., 2005; Practice Committee of the ASRM, 2012; Carp, 2014; Ferriani *et al*., 2018; ESHRE Guideline Group on RPL *et al*., 2023; Homer, 2019; Regan *et al*., 2023; Dimitriadis *et al*., 2020).

Chronic endometritis (CE) is defined as chronic inflammation of the endometrial lining. Patients with CE are often asymptomatic, though they may present with symptoms such as pelvic pain, dyspareunia, abnormal vaginal bleeding, or vaginal discharge (Romero *et al*., 2004). Studies report a prevalence of CE among RPL patients ranging from 13% to 56% (Bouet *et al*., 2016; Zargar *et al*., 2020; Oliveira *et al*., 2023). Although the gold standard for diagnosing CE is the histological identification of plasma cells in the endometrial stroma through immunohistochemical evaluation, hysteroscopic diagnosis also has reasonable sensitivity and specificity when endometrial biopsy is not readily available (Liu *et al*., 2020).

The pathophysiology of CE is not fully understood, but its relationship with the endometrial microbiome is widely accepted, as evidenced by the effectiveness of antibiotic treatment in reducing stromal infiltration (Kitaya *et al*., 2018; Kimura *et al*., 2019; Pirtea *et al*., 2021). It is believed that plasma cell infiltration of the endometrium may affect endometrial receptivity and result in RPL (Bouet *et al*., 2016). Several studies have evaluated the efficacy of antibiotic regimens in treating CE and their impact on pregnancy outcomes in women with RPL (Liu *et al*., 2022; Strug *et al*., 2024).

The aim of this review article is to evaluate the relationship between CE and RPL, considering its pathophysiological mechanisms, diagnosis and pregnancy outcomes after treatment.

## METHODS

A comprehensive, non-systematic search on the association between RPL and CE was conducted in the PubMed, Cochrane, and SciELO databases, considering its pathophysiological mechanisms, diagnosis and pregnancy outcomes after treatment. The following terms were used as keywords: (“chronic endometritis” OR “endometrial inflammation” OR “subclinical endometritis”) AND (“recurrent pregnancy loss” OR “recurrent miscarriage” OR “pregnancy outcomes”).

The electronic search was conducted in August 2024, and only articles published in English were included. Due to the limited data available in the literature, various types of studies were accepted (cohort studies, cross-sectional studies, clinical trials, and meta-analyses) with no restriction on publication date. Additionally, the reference lists of the identified articles were reviewed to expand the selection of relevant studies.

Two independent reviewers (ECFO and FCCA) read and analyzed the selected articles.

## RESULTS

Initially, 132 studies were identified in PubMed, 16 in Cochrane and none in SciELO, After screening by titles and abstracts, followed by reading the potentially eligible articles in full, 15 studies were included in this review. Observational cohort and cross-sectional studies were prioritized. No randomized clinical trials were found. The main reasons for exclusion of articles were: duplication, lack of data on the outcomes of interest and type of publication (such as narrative reviews without quantitative data and case reports).

To facilitate the discussion, the results were categorized in pathophysiological mechanisms, diagnosis and treatment and pregnancy outcomes.

### Pathophysiological mechanisms

The correlation between the endometrial microbiome and CE is well established in the literature. The bacteria most frequently associated with CE are those that colonize the female urogenital tract, such as *Streptococcus* spp., *Escherichia coli, Enterococcus faecalis, Klebsiella pneumoniae, Staphylococcus* spp., *Corynebacterium*, and *Mycoplasma/Ureaplasma* spp. However, not all women who have these bacteria in their endometrial flora develop the disease, suggesting that the interaction with the local immune system is crucial (Kimura *et al*., 2019; Puente *et al*., 2020). This imbalance can increase the production of pro-inflammatory cytokines, altering processes such as cell migration, proliferation, and apoptosis, thus impairing embryonic implantation and pregnancy maintenance (Kimura *et al*., 2019; Puente *et al*., 2020).

CE also affects endometrial genomic expression. During the secretory phase, there is an overexpression of anti-apoptotic genes, as well as nuclear markers associated with proliferation (Ki-67) and ovarian steroid receptors. On the other hand, the expression of genes associated with embryonic receptivity and decidualization is reduced (Kitaya *et al*., 2018). The overexpression of estrogen and progesterone receptors may lead to a loss of the endometrial implantation window, hindering the synchronization between the endometrial cycle and the embryo’s arrival in the uterine cavity (Kitaya *et al*., 2018).

### Diagnosis

CE can be suspected during hysteroscopy by identifying at least one of the following criteria: increased endometrial vascularization, stromal edema, a “strawberry-like” mucosa (hyperemia with scattered white spots), and the presence of endometrial micropolyps < 1 mm. The diagnostic specificity of outpatient hysteroscopy is 80%, while sensitivity is only 40% (Bouet *et al*., 2016). However, the accuracy of the exam depends on the examiner’s experience and the type of findings, with micropolyps being the criterion with the highest positive and negative predictive values (increased endometrial vascularization, stromal edema, a “strawberry-like” mucosa (hyperemia with scattered white spots), and the presence of endometrial micropolyps < 1 mm, respectively) (Kimura *et al*., 2019; Puente *et al*., 2020). Thus, hysteroscopy is extremely useful, especially when endometrial biopsy is not available. However, it should ideally be complemented with a histopathological sample (Bouet *et al*., 2016; Kimura *et al*., 2019).

The histopathological detection of plasma cells in the endometrial stroma using the CD-138 immunohistochemical marker is the most accurate method for diagnosing CE (Pirtea *et al*., 2019). However, the diagnosis of CE still lacks standardization in several aspects (Kitaya *et al*., 2018; Kimura *et al*., 2019; Pirtea *et al*., 2019). There is no consensus on the cutoff for the number of plasma cells required for diagnosis, nor on the dilution of CD-138 to be used (Pirtea *et al*., 2019). Some authors argue that the identification of even a single plasma cell is sufficient for diagnosis (Bouet *et al*., 2016; Kimura *et al*., 2019). However, monoclonal antibodies used to mark CD-138 in plasma cells can also react with endometrial epithelial cells, leading to false positives and overdiagnosis (Bouet *et al*., 2016). A meta-analysis of 13 studies, aimed at determining a cutoff point indicative of a worse prognosis in women with RPL, identified that a plasma cell count above 4-6 cells/hpf is associated with an increased risk of further miscarriages (Rimmer *et al*., 2021).

Regarding endometrial sample collection, there is also no consensus on the optimal phase of the menstrual cycle for biopsy, the macroscopic characteristics of the best site for biopsy, the number of samples required, or the depth of the biopsy (Kitaya *et al*., 2018; Kimura *et al*., 2019; Rimmer *et al*., 2021).

The method of obtaining the endometrial sample, quality control of the CD-138 used, and the definition of the minimum number of plasma cells required are parameters that need to be standardized to establish guidelines for the diagnosis, treatment, and monitoring of CE (Bouet *et al*., 2016; Kitaya *et al*., 2018). The use of next-generation sequencing of the 16S ribosomal subunit and/or real-time polymerase chain reaction are viable tests that can identify microorganisms in the uterine cavity, aiding in elucidating the causal relationship with chronic endometritis and in developing appropriate treatments (Kitaya *et al*., 2018). Additionally, metagenomics may help not only to eliminate pathogenic flora but also, in the future, to develop methods for colonizing the uterine cavity with flora that promotes reproductive success (Puente *et al*., 2020). The diagnostic criteria for CE are summarized in Table 1.

**Table 1:** Diagnostic criteria and treatment for chronic endometritis according to different studies.

Study and year of publication	Hysteroscopy performance time	Number of plasma cells considered for diagnostic criteria	Antimicrobial treatment
Bouet *et al*., 2016	Follicular phase (between days 6 and 12)	≥ 5 plasma cells per 10 HPFs	Doxycycline 100 mg orally twice a day for 14 days
Zargar *et al*., 2020	3-5 days after menstruation; HC staining evaluation using anti sundecan-1 (CD 138)	≥ 5 plasma cells per 20 HPFs	Doxycycline 100 mg orally twice a day for 21 days
Rimmer *et al*., 2021	Secretory stage (certified by transvaginal ultrasound scan between days 4-14 post positivity of LH strips)	ROC analysis suggests an overall good performance for CD138+ as a diagnostic test with scores above a cutoff value of 4-6 cells/HPF	Not evaluated
Gay *et al*., 2021	Not described	≥ 1 plasma cell (s) per HPFs	Antibiotic adapted to antibiogram, or doxycycline (100 mg twice a day) plus metronidazole (500 mg twice a day) orally for 14 days
Takimoto *et al*., 2023	Mid-luteal phase, confirmed by transvaginal ultrasound and the last menstrual cycle	Liu’s method (Plasma cell count > 5.15/10 mm^2^), McQueen scores and plasma cell count/10 mm^2^	Not evaluated
McQueen *et al*., 2014	Not described	H&E >1 plasma cell in the whole section	Ofloxacin 400 mg + metronidazole 500 mg orally twice a day for 14 days Or doxycycline +/- metronidazole or ciprofloxacin + metronidazole
Cicinelli *et al*., 2014	Follicular phase of menstrual cycle	> 1 plasma cell/ HPFs	Gram-negative: ciprofloxacin 500 mg twice/day for 10 d. Gram-positive: amoxicillin + clavulanate 1 g twice/day for 8d. Mycoplasma or U urealyticum: josamicine: 1 g twice/day for 12d. Negative culture: ceftriaxone 250 mg i.m. + doxycycline 100 mg orally twice/day for 14d + metronidazole 500 mg orally twice/day for 14 d (CDC guidelines)
McQueen *et al*., 2015	Not described	1-5 plasma cells per HPF, or discrete clusters of <20 plasma cells by CD138 staining.	Doxycycline 100 mg twice a day for 14 or 21 days according to physician preference

HPF: high power fields; LH: luteinizing hormone.

### Antibiotic choice for chronic endometritis and pregnancy outcomes after treatment

CE is a condition frequently associated with RPL and recurrent implantation failures, necessitating the exploration of effective treatment regimens. Several studies have investigated different therapeutic approaches, focusing mainly on antibiotic use, and have evaluated the efficacy of each regimen in terms of resolving the condition and impacts on reproductive outcomes.

A study conducted by McQueen *et al*. focused on women with early RPL and/or fetal demise. A total of 395 women were analyzed, including 285 with a history of RPL, 57 with fetal demise, and 53 with both conditions. The prevalence of chronic endometritis in the overall sample was 9% (35/395). Treatment was carried out with ofloxacin 400 mg and metronidazole 500 mg twice a day for two weeks. Nine women were treated with a different regimen: doxycycline, doxycycline plus metronidazole, or ciprofloxacin plus metronidazole. All 35 women received treatment, and 31 underwent follow-up endometrial biopsy to assess cure. Of these, 23% (7/31) still showed infection (all previously treated with ofloxacin and metronidazole). Two of these underwent another course of antibiotics and had documented resolution. The remaining five did not take another antibiotic course but had a repeat hysteroscopy 10 weeks later, which confirmed resolution. Therefore, cure with a single antibiotic course was achieved in 94% of those who had follow-up (29/31). Considering two antibiotic courses, 100% of the follow-up tests showed resolution of chronic endometritis. There was no statistically significant difference in live birth rates between women with treated CE and those without CE (88% *vs*. 74%, *p*=0.215). During the follow-up period, both groups showed a significant increase in live birth rate per pregnancy; however, in the treated CE group, the improvement was more pronounced (from 7% to 56% *vs*. from 15% to 59%, respectively; *p*=0.04) (McQueen *et al*., 2014).

A retrospective cohort study by Cicinelli *et al*. involved 360 women with RPL, of whom 208 were diagnosed with CE through hysteroscopy. Cultures of endometrial samples were also performed, and antibiotic treatment was tailored based on antibiogram results: ciprofloxacin was used for gram-negative bacteria, amoxicillin plus clavulanate for gram-positive bacteria, josamycin for mycoplasma and *Ureaplasma urealyticum*, and a combination of ceftriaxone, doxycycline, and metronidazole for negative cultures, in accordance with CDC guidelines. Treatment normalized hysteroscopy and culture in 71.8% of women who had abnormalities in both tests. One year after treatment, the live birth rate was significantly higher in groups with normalized hysteroscopy (78.4% in the previously culture-positive group [n=80] and 50% in the culture-negative group [n=8]), compared to those with persistent CE on hysteroscopy (30% in previously culture-negative [n=15] and 17.5% in culture-positive [n=7]) (Cicinelli *et al*., 2014).

In a retrospective study by Gay *et al*., 42 women with RPL were analyzed, with 22 diagnosed with CE by immunohistochemistry. Treatment was based on antibiogram results or doxycycline and metronidazole for 14 days. There was a significant increase in live birth rates in treated women (85%), compared to untreated women (44%) or those without a CE diagnosis (40%) (*p*=0.032). Additionally, there was a significant reduction in miscarriage rates in the treated group (Gay *et al*., 2021).

Lastly, HogenEsch *et al*. evaluated women undergoing infertility treatment who had endometrial sampling. Doxycycline was the first-line antibiotic in over 75% of cases, followed by regimens such as ciprofloxacin plus metronidazole or ofloxacin plus metronidazole in persistent cases. CE was resolved in 68.5% of cases after the first treatment, increasing to 88.3% after up to two cycles of antibiotics. The live birth rate was significantly lower in untreated CE cases compared to those with resolved CE (HogenEsch *et al*., 2023).

In summary, antibiotic regimens vary in their efficacy but show considerable success in resolving chronic endometritis, which is reflected in improved reproductive outcomes. Treatment choice should consider individual microbial response and the persistence of inflammation, with adaptation based on antibiograms when possible. Diagnostic criteria and treatment for chronic endometritis according to different studies are described in Table 1.

### Limitations

This narrative review included a range of studies, from meta-analyses to other narrative reviews and case series. Several limitations were identified, including small sample sizes and significant methodological and outcome heterogeneity, which hinder direct comparisons across studies. The lack of standardized diagnostic criteria for CE contributes to substantial variations in its reported prevalence and poses challenges in assessing treatment success.

In studies evaluating treatment efficacy, distinguishing true resolution of the condition from false-negative findings during post-treatment follow-up remains problematic (Liu *et al*., 2022). This issue highlights the need for more robust diagnostic tools and standardized protocols to improve the accuracy and comparability of research findings.

The absence of randomized controlled trials further limits the evidence base, leaving room for potential biases in assessing the relationship between CE and RPL. These challenges underscore the importance of future studies designed with larger cohorts, rigorous methodology, and consistent diagnostic and treatment criteria to advance understanding and clinical management of CE (El Hachem *et al*., 2017).

## SUMMARY AND CONCLUSIONS

CE appears to be a significant but often underdiagnosed contributor to RPL. However, the absence of randomized controlled trials represents a major limitation in establishing causal inferences between CE treatment and improved reproductive outcomes. Therefore this narrative review highlights significant methodological variability across studies investigating CE in the context of RPL. Advancements in diagnostic techniques, including the use of CD-138 markers and molecular tests, have improved the accuracy of CE identification, although standardization remains a challenge. Evidence suggests that effective antibiotic treatment not only resolves CE but also enhances pregnancy outcomes in affected women. Further research, particularly randomized controlled trials, is needed to refine diagnostic criteria and optimize treatment protocols. Addressing these gaps will be critical to improving care for women with RPL associated with CE.

### Key points:

- Diagnostic hysteroscopy can be useful for identifying CE based on visual findings (e.g., edema and endometrial micropolyps). However, specificity and sensitivity vary, and it is ideally complemented by biopsy.
- The identification of plasma cells using the CD-138 marker is the most accurate method for diagnosing CE. Standardization is still needed for the number of plasma cells considered diagnostic and for the quality of CD-138.
- Tests such as next-generation sequencing and real-time PCR can identify microorganisms and aid in the development of appropriate treatments.
- Antibiotic regimens have shown efficacy in reducing CE and have a positive impact on pregnancy outcomes in women with RPL.
